# An Unusual Tumor of the Ear: Pilomatricoma in a Middle-Aged Woman

**DOI:** 10.1155/2019/4975216

**Published:** 2019-12-14

**Authors:** Akira Inagaki, Yoriko Yamashita, Yusuke Mori, Erika Takeuchi, Kyosuke Asaoka, Shingo Murakami

**Affiliations:** ^1^Department of Otolaryngology, Head and Neck Surgery, Graduate School of Medical Sciences, Nagoya City University, Nagoya 467-8601, Japan; ^2^Department of Experimental Pathology and Tumor Biology, Graduate School of Medical Sciences, Nagoya City University, Nagoya 467-8601, Japan

## Abstract

Pilomatricoma is a benign tumor arising from hair follicle matrix cells, presenting as an asymptomatic, firm, slow growing, mobile, superficial skin nodule typically in children. This lesion with an atypical clinical presentation is frequently misdiagnosed as other skin lesions and even as malignant entities regardless of detailed cytological, imaging examinations; the site of occurrence is one of the keys to accurate diagnosis. Here, we present a case of pilomatrixoma involving the ear, the cymba conchae of the auricle, which is an extremely rare site for the lesion in a 52-year-old woman. The present case suggests that this benign tumor needs to be included in the differential diagnosis in patients who present with an atypical auricular lesion.

## 1. Introduction

Pilomatricoma is a benign tumor arising from hair follicle matrix cells. It is a relatively common entity, accounting for about 1% of all benign skin lesions [1]. It occurs frequently in the pediatric population and has a slight female predilection [2]. Typically, it presents as an asymptomatic, firm, slow growing, mobile, superficial skin nodule of 0.5 to 3 cm in diameter [1, 3]. However, pilomatricomas with an atypical shape are frequently misdiagnosed as other skin lesions and even as malignant entities [4, 5]. The site of occurrence is one of the keys to accurate diagnosis. In children, the head and neck are the sites most frequently involved [2], particularly the neck and periauricular regions [1]. Although pilomatricomas are common in adjacent areas, the auricle is a rare site for this lesion; the English literature contains two cases in infants but, to the best of our knowledge, none have been reported in adults. Here, we present a case of pilomatrixoma involving the ear in an adult patient.

## 2. Case Presentation

**Figure 1:**
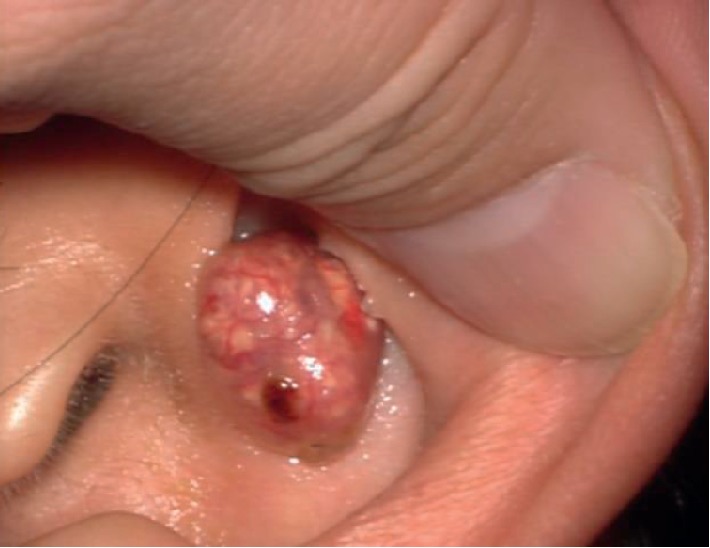
Photograph showing a lesion located on the cymba conchae at the second visit 4 weeks later. The color of the tumor under the semitransparent overlying skin has changed from red to yellowish white.

**Figure 2:**
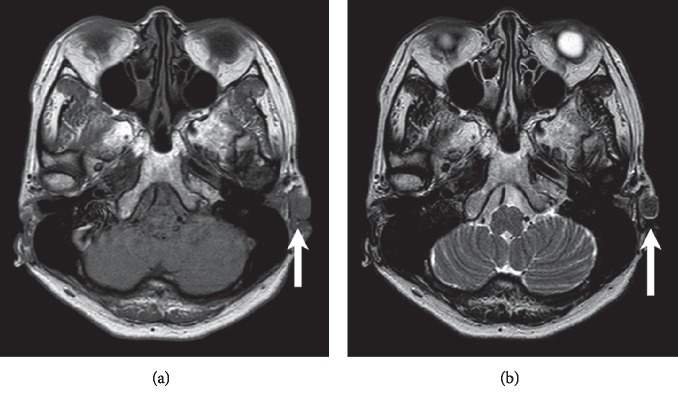
Magnetic resonance imaging revealing a solitary, well-circumscribed 11 × 10 mm tumor that was isointense on T1-weighted (a) and T2-weighted (b) images. There was no diffusion restriction on diffusion-weighted imaging (not shown).

**Figure 3:**
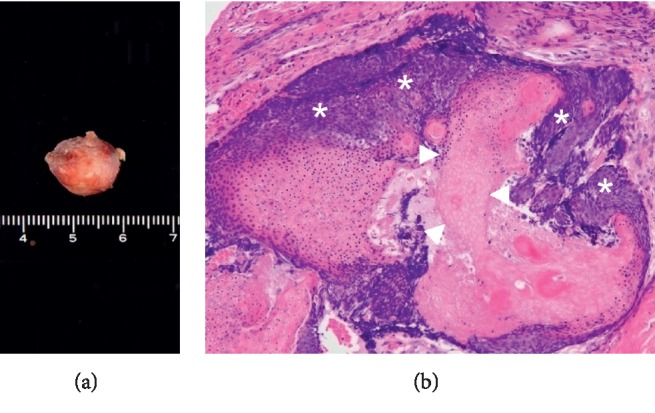
The image of the excised tumor. (a) A well-circumscribed encapsulated nodule containing an irregularly shaped tumor was removed. (b) Tissue section showing islands of basaloid cells (asterisks) and shadow cells (arrowheads), clearly indicating a diagnosis of pilomatricoma (hematoxylin and eosin staining).

A 52-year-old woman presented to our clinic with the complaint of a nodule on the left auricle. She had a history of adenomyomatosis of the gallbladder but was otherwise healthy. Two weeks earlier, she had visited a local otolaryngologist who treated the nodule with centesis, which resulted in temporary shrinkage of the lesion but rapid regrowth thereafter. On the initial visit to our clinic, the nodule was approximately 1 cm in size. It was mobile, red, tense, cystic, and located in the cymba conchae of the left auricle. However, 4 weeks later, the redness had disappeared, and a yellowish white, irregular, semitransparent nodule with thin red-edged protrusions had emerged beneath the thin overlying skin (Figure 1). We also noticed that the lesion was larger at the second visit, which prompted a request for magnetic resonance imaging (MRI) of the head and neck to rule out malignancy. MRI revealed a solitary, well-demarcated 11 × 10 mm lesion isointense on both T1-weighted (Figure 2(a)) and T2-weighted (Figure 2(b)) images, and there was no diffusion restriction on diffusion-weighted MRI, suggesting a benign entity. Excision with 2 mm margins was performed, and a well-circumscribed encapsulated nodule containing a yellowish white, irregularly shaped tumor with a thin layer of overlying skin was removed (Figure 3(a)). The skin defect was covered with a small skin flap. Histological examination revealed lobulated structures containing clusters of basophils and abundant shadow/ghost cells, clearly indicating a diagnosis of pilomatricoma (Figure 3(b)). Occasional keratins and foreign body giant cells were noted, but no calcification or ossification was observed. The postoperative course was uneventful, and there has been no recurrence during 12 months of follow-up.

## 3. Discussion

The auricle is a rare site for pilomatricoma, especially in adulthood. There have been two reports of auricular pilomatricoma in the pediatric population in the English-language literature (one in a 3-year-old boy and the other in a 4-year-old boy, but none in adults). A review of 205 cases of pilomatrixoma that included patients of all ages found that the preauricular area was the second most frequent site involved (17.1%) but did not mention any cases involving the auricle [1].

As with most cutaneous sites, the auricle can harbor various lesions, the most common of which is squamous cell carcinoma [6]. Moreover, there are tumors that occur almost exclusively on the auricle, such as benign and malignant tumors, originating from the ceruminous gland [7]. Pilomatricoma, also known as calcified epithelioma of Malherbe, is unique as its hallmark is internal calcification [2]. In one case series, histopathological examination identified either calcification or ossification in 81% of the tumors [3]. However, diagnosis of pilomatricoma is not always straightforward and may even be challenging, with only 28.9% of cases reported to be accurately diagnosed preoperatively [8].

Some of the misdiagnoses can be ascribed to inflammation. A histological examination of 346 cases identified inflammation in 40.8% of cases [8]. Pain and tenderness was reported in about 20% of cases; consequently, the overlying skin can have a reddish or bluish hue [8], which makes it difficult to inspect or palpate for the characteristic features of pilomatricoma, namely, a hard, irregular, subcutaneous nodule that is freely movable under the overlying skin [9], as was the case in our patient at the initial visit.

Change in the cytomorphological characteristics of the lesion as it progresses may also contribute to misdiagnosis. The cells in a pilomatricoma are typically arranged in a circular configuration, with basaloid cells on the periphery that facilitate transition to shadow cells in the center and undergo dystrophic calcification with time. Basaloid cells are predominant in the early stages; however, in the later stages, the proportion of shadow cells with accompanying calcification becomes greater [10]. Pilomatricomas are more likely to be resected at an early stage [2] when internal calcification is limited, which may pose some difficulties in terms of the differential diagnosis, as reported previously [9].

On cytological examination, partial presentation of the components of a pilomatrixoma, namely, epithelial sheets containing small basaloid and shadow cells, as well as foreign body giant cells and calcified ghost cells, may lead to misdiagnosis. One study found that up to 60% of pilomatricomas (39 of 66 cases) were misdiagnosed by fine needle aspiration cytology [2]. This is because smears containing a predominance of basaloid cells are easily misdiagnosed as malignancy due to their high nuclear/cytoplasmic ratio, and those with a predominance of squamous cells, shadow cells, and foreign body giant cells may be misdiagnosed as squamous cell carcinoma [4]. In fact, a case was reported in which an unnecessarily wide excision was performed in a patient with a pilomatrixoma that had been incorrectly diagnosed as a malignant lesion [5].

In contrast to most other nodules, diagnostic imaging is of uncertain value for pilomatrixoma. Computed tomography (CT) identified internal calcification in 81% of cases [11], while ultrasonography visualized the internal echogenic foci in 93% [12]. On MRI, these lesions were isointense on T1-weighted images in 67% of cases, and hyperintense or hypointense on T2-weighted images in 76.2%. Pilomatrixoma was also found to demonstrate abnormally high uptake of fluorodeoxyglucose on positron emission tomography (PET)/CT [5]. However, the diagnostic value of these imaging features is likely limited. For example, one study showed that only 1 (3.1%) of 32 cases was accurately diagnosed as pilomatricoma by CT, MRI, or PET/CT [2]. Furthermore, in another study, only 13.3% of pilomatricomas were accurately diagnosed in pediatric patients by CT, MRI, or ultrasonography [13].

## 4. Conclusion

Auricular pilomatricoma can pose a diagnostic pitfall for the reasons outlined above. The auricle is a rare site for pilomatricoma in adults. However, despite the diagnostic challenges of this entity, it would be worthwhile to include this benign tumor in the differential diagnosis in patients who present with an atypical auricular lesion irrespective of whether the potential diagnosis is benign or malignant.
